# Gut microbiota changes in the extreme decades of human life: a focus on centenarians

**DOI:** 10.1007/s00018-017-2674-y

**Published:** 2017-10-14

**Authors:** Aurelia Santoro, Rita Ostan, Marco Candela, Elena Biagi, Patrizia Brigidi, Miriam Capri, Claudio Franceschi

**Affiliations:** 1 Department of Experimental, Diagnostic and Specialty Medicine (DIMES), Alma Mater Studiorum-University of Bologna, Via San Giacomo 12, 40126 Bologna, Italy; 2 Interdepartmental Centre “L. Galvani” (CIG) Alma Mater Studiorum-University of Bologna, Via San Giacomo 12, 40126 Bologna, Italy; 3 Department of Pharmacy and Biotechnology (FABIT), Alma Mater Studiorum-University of Bologna, Via Belmeloro 6, 40126 Bologna, Italy; 4 Institute of Neurological Sciences (IRCCS), Via Altura 3, 40139 Bologna, Italy

**Keywords:** Gut microbiota, Aging, Centenarians, Gut–brain axis, Host genome

## Abstract

The gut microbiota (GM) is a complex, evolutionarily molded ecological system, which contributes to a variety of physiological functions. The GM is highly dynamic, being sensitive to environmental stimuli, and its composition changes over the host’s entire lifespan. However, the basic question of how much these changes may be ascribed to variables such as population, diet, genetics and gender, and/or to the aging process per se is still largely unanswered. We argue that comparison among studies on centenarians—the best model of healthy aging and longevity—recruited from different geographical areas/populations (different genetics and dietary habits) can help to disentangle the contribution of aging and non-aging-related variables to GM remodeling with age. The current review focuses on the role of population, gender and host genetics as possible drivers of GM modification along the human aging process. The feedback impact of age-associated GM variation on the GM–brain axis and GM metabolomics is also discussed. We likewise address the role of GM in neurodegenerative diseases such as Parkinson’s and Alzheimer’s, and its possible therapeutic use, taking advantage of the fact that centenarians are characterized by an extreme (healthy) phenotype versus patients suffering from age-related pathologies. Finally, it is argued that longitudinal studies combining metagenomics sequencing and in-depth phylogenetic analysis with a comprehensive phenotypic characterization of centenarians and patients using up-to-date omics (metabolomics, transcriptomics and meta-transcriptomics) are urgently needed.

## The study of the human gut microbiota: methodological aspects

The human gut microbiota (GM) is a highly diverse ecosystem made up of trillions of bacteria populating the gastrointestinal tract. This niche establishes a complex, multi-species apparatus in which every occupant plays a role and modulates its own activity in response to signals coming from inside and outside the human host [1]. The composition of the GM is affected by a plethora of individual, population and environmental variables, e.g., age, gender, genetic background, biography (type of delivery, breastfeeding or formula feeding, use of antibiotics), immuno-biography (lifelong immunological experience) and geography (ethnicity, cultural habits, nutrition). These factors over a lifetime impinge on the GM, resulting in huge variability and heterogeneity of this ecosystem in human beings. This adaptive nature of the GM is functional to calibrating the immune and metabolic pathways in response to individual needs, and has a profound impact on health and disease. Indeed, the GM has emerged as a dynamic community able to adapt its composition and functionality to the varying conditions in which the human host lives to meet the changing demands of host metabolism [2]. Thus, a healthy adult GM structure is properly defined as a set of many possible configurations which, even when differing in composition, share a comparable degree of diversity and evenness (meaning the number of species with an equal distribution in the ecosystem), and the ability to preserve the homeostasis of the human host [3]. In this elaborate scenario, the most informative approach for understanding the role of the GM in its lifelong maintenance of host homeostasis would clearly be by longitudinal studies monitoring individuals over time (years and decades) to identify and follow the specific trajectories of their age-related GM modifications. To date, this kind of analyses has not been possible because attention towards the GM is quite a recent development, while the most reliable and robust longitudinal studies have not collected stool samples across the full life span of individuals. Hopefully, new life-long longitudinal studies or continuations of existing ones will cover this gap.

At present, the best way of grasping the adaptive pattern of human GM as humans age is represented by cross-sectional studies embracing a wide age range in well-defined populations that are relatively homogeneous in genetics and lifestyles. Inclusion of “extreme phenotypes”, i.e., individuals who are at the extreme ends of a trait distribution (healthy subjects versus patients suffering from diseases), can help in identifying specific signatures within overall age-related trajectories, regarding genetics, epigenetics, metabolomics, and including metagenomics, among other things [4–8]. Such is the case of centenarians who represent a clearly defined and highly informative “super-control” group, since, unlike younger controls, most of them achieved their remarkable age by avoiding or perhaps postponing major age-related diseases. The strategy of focusing on individuals from well-defined populations and including the “extreme phenotypes” such as centenarians increases one’s power to identify physiological age trajectories, including the last 20–30 years of human life which are usually neglected [9]. Comparison between data sets obtained from different populations will allow us to disentangle changes related to specific genetic or lifestyle habits, including diet, from changes related to the aging process itself.

### The model of centenarians

Centenarians represent the best model of “successful” aging showing a lower incidence of chronic illness, a reduction of morbidity and an extension of health span in comparison to octogenarians and nonagenarians from the same cohort [10, 11]. Thus, the study of the GM of exceptionally long-lived individuals is providing insights into how the GM successfully adapts in an extremely long lifespan to the progressive age-related environmental (lifestyle, diet, etc.) and endogenous changes, contributing to the maintenance of metabolic and immunological homeostasis and promoting survival [1, 8].

Human longevity has a strong familial and genetic component [12, 13]. Data from different populations have shown that relatives (parents, siblings and offspring) of long-lived subjects have a significant survival advantage, a higher probability of being or becoming long-lived and a lower risk of undergoing major age-related diseases [14–17]. Family genealogy data from Sardinian centenarian women have confirmed that maternal longevity is associated with lower infant mortality in offspring [18] suggesting that parents/mothers who will later become centenarians very likely adopt healthier lifestyles for their children. Considering that the study of centenarians has some obvious limitations (rarity, lack of an age-matched control group and frailty related to extreme age), centenarians’ offspring, representative of the elderly age bracket whose lifestyle can still be modified to attain better health, may provide a useful model to study both genetic and environmental/lifestyle determinants of healthy aging [14].

Starting from observation of the profound changes in immune responses with age (immunosenescence, i.e., the overall age-related remodeling of the immune system [19]) and taking into account the increasing amount of experimental data on genetics, proteomics, epigenetics, metabolomics, glycomics, etc. [20], one may conceptualize the aging process as a continuous lifelong remodeling of the whole human organism [21]. The exceptional phenotype of centenarians has been revealed as unexpectedly complex and very dynamic, being a unique mixture of adaptive robustness and accumulating frailty [21–26] resulting from the ability of the centenarian’s organism to respond/adapt to damaging stimuli.

According to the dynamics of world population aging, a lifelong approach including the last decades of life is extremely important if we are to understand the basis of the longevity process, considering that the oldest-old are the fastest growing segment of the population in many countries. It is also interesting to note that the birth cohort is crucial in the health outcome of long-lived people. A comparison of two Danish cohorts born 10 years apart (1905 and 1915) showed that the younger cohort had longer survival and scored significantly better on both cognitive tests and the activities of daily living scale than the cohort born in 1905, despite being 2 years older at the time of assessment. This finding suggests that more people are living to older ages with better overall functioning [27].

Demographic projections suggest that there will be 3.7 million centenarians across the globe in 2050. In particular, China is expected to have the largest centenarian population, followed by Japan, the United States, Italy and India. In this scenario, the global number of persons aged 80 or over is projected to increase from 125 million in 2015 to 434 million in 2050 with a dramatic hike of the resources needed to care for them [28].

## Gut microbiota from birth to 100 years and beyond

The programming of immune response and metabolic pathways is heavily influenced by the interaction between the human organism and its GM starting from infancy. This bidirectional relationship in early life has a profound impact on health and disease in later life.

A very recent paper has proposed that the progressive process of microbial colonization of the human ecosystems may be initiated in utero by the microbial populations of the maternal placenta and amniotic fluid which share some features with the microbiota detected in infant meconium [29]. Moreover, during vaginal delivery, a considerable inoculum of maternal intrauterine microbes is received by the neonate and, after birth, neonatal gut colonization is continued by microbes present in maternal milk and feces, with human milk factors (e.g., complex polysaccharides and antibodies) selectively promoting the growth of mutualistic microbial partners. Thus, antibiotic exposure during pregnancy, cesarean section delivery, postnatal antibiotic administration, and formula feeding may all alter the early intestinal microecology, and these factors have been associated with the risk of disease in later life [30–34]. These findings reveal that the aging process could also depend on early stimuli and experiences that may exert long-term effects. To the best of our knowledge, no studies have correlated the physiological and/or pathological phenotype of elderly and extremely old individuals with these initial events shaping the early GM establishment. For instance, in centenarian databases no data are available on the mode of delivery, breast- or other types of feeding (wet nurse, animal milk, etc.), nutrition and hygienic conditions in the early years of life. Historical anthropology studies could shed light on these points.

Starting from life in utero, the gastrointestinal tract is colonized by a wide range of bacteria of maternal, dietary and environmental origin, which, after assembling themselves into a highly interconnected bacterial community, co-operate in several vital host functions, including nutrient digestion and absorption, immune function, as well as the development of an appropriate stress response. This close symbiotic relationship makes humans inter-dependent “meta-organisms” [35, 36], where the commensal bacteria function as a metabolic and endocrine organ [37] and, in turn, the human immune system has properly evolved to control the physiological life-long low-grade inflammatory response triggered by the GM.

The human GM metacommunity is estimated to consist of over 1000 different microbial species [38] belonging to 5 predominant phyla: *Firmicutes* and *Bacteroidetes* followed by *Actinobacteria*, *Verrucomicrobia* and *Proteobacteria* [39, 40]. As previously discussed, the GM is a malleable ecosystem, being able to adapt its phylogenetic and functional profile to changes in diet, environment, lifestyle, antibiotic treatments and stress. In a mutualistic context, this plasticity is functional to optimizing the metabolic and immune performance of the host in response to environmental and physiological changes, preserving physiological homeostasis and health status [41].

The human GM is a complex and dynamic environment, which undergoes profound life-long remodeling, sometimes with a concrete risk of maladaptive changes. Indeed, in certain circumstances the age-related pathophysiological changes in the gastrointestinal tract, modification of lifestyle, nutrition [42] and behavior, as well as immunosenescence and “inflammaging” (the chronic low-grade inflammatory status typical of the elderly, [23]) strongly impact on the GM, eventually forcing maladaptive variations [43]. Inflammation, in particular, may result in a higher level of aerobiosis and production of reactive oxygen species that inactivate the strict anaerobic *Firmicutes*, while allowing a bloom of facultative aerobes, as frequently observed in the elderly [41]. These microorganisms (i.e., *Enterobacteriaceae*, *Enterococcaceae*, *Staphylococcaceae*), generally called “pathobionts”, can prosper in an inflamed gut as they are relatively oxygen tolerant, getting the better of mutualistic symbionts and further supporting inflammation [44]. On the other hand, these age-related GM changes can compromise the host immune homeostasis in favor of a pro-inflammatory profile creating a vicious inflammatory circle and may contribute to the progression of diseases and frailty in the elderly [45–47]. Frailty has been negatively associated with GM diversity [48] and *Eubacterium dolichum* and *Eggerthella lenta* have been found to be more abundant among frail individuals, while *Faecalibacterium prausnitzii* was less abundant, thus identifying a GM signature of frailty [48]. A very recent publication has demonstrated that germ-free mice are protected from inflammaging [49]. When these mice are co-housed with old, but not young, mice the levels of pro-inflammatory cytokines in the blood increase together with intestinal permeability and macrophage dysfunction [49]. On the whole, these data prove that age-related dysbiosis is responsible for the age-related increase in systemic inflammation. Thus, pursuing a wholesome and adaptive GM trajectory during aging is dramatically emerging as a key factor in the achievement of healthy aging and maintenance of host homeostasis [50].

The comparison of GM among young adults, the elderly, and centenarians has highlighted that the mutualistic changes in the composition and diversity of the gut ecosystem do not follow a linear relation with age, remaining highly similar between young adults and 70-year-olds and markedly changing in centenarians. Thus, the GM seems to rest in a stable state from the third to the seventh decade of life [51], while after 100 years of symbiotic association with the human host, it shows a profound, and possibly adaptive, remodeling. Further analyses are needed to fill in the age gap between 70 and 100 years of age and complete the re-construction of age-related GM modifications.

Centenarians stand out as a separate population, their GM showing high diversity in terms of species composition (Table 1) [52]. *Bacteroidetes* and *Firmicutes* still dominate the GM of centenarians, but *Firmicutes* subgroups go through specific changes with a decrease in the contributing *Clostridium* cluster XIVa, an increase in *Bacillus* species, and a rearrangement of the *Clostridium* cluster IV composition. Several butyrate producers (*Ruminococcus obeum* et rel., *Roseburia intestinalis* et rel., *Eubacterium ventriosum* et rel., *Eubacterium rectale* et rel., *Eubacterium hallii* et rel., *Papillibacter cinnamovorans* et rel., *and Faecalibacterium prausnitzii* et rel.) were found in lower amounts, while others (*Anaerotruncus colihominis* et rel. and *Eubacterium limosum* et rel.) increased in centenarians, suggesting the existence of bacteria characteristic of longevity [51].

**Table 1:** Changes to the index of GM diversity in centenarians according to different papers

Diversity	Change in centenarians according to				
	Biagi et al. [51]	Biagi et al. [1]	Wang et al. [61]	Kong et al. [52]	Odamaki et al. [70]
Simpson Reciprocal Index of Diversity	**↓**	**↑**			
Alpha diversity (Chao Index)		**↑**	**↑**	**↑**	**↑**
Shannon Index		**↑**	**=**	**↑**	**↑**

The GM of centenarians is enriched in facultative anaerobe bacteria mostly belonging to *Proteobacteria* which have been redefined as “pathobionts” because, in some circumstances, e.g., inflammation, they may escape surveillance, prevail over mutualistic symbionts and induce pathology [44, 53]. The age-related remodeling of GM (i.e., proliferation of opportunistic *Proteobacteria* at the cost of symbiont *Firmicutes* and *Bacteroidetes*) may contribute to inflammaging and/or is affected by the systemic inflammatory status in a sort of self-sustaining loop. Indeed, the changes in GM profile observed in centenarians correlate with an increase in pro-inflammatory cytokines in the peripheral blood. In particular, these exceptionally long-lived subjects show high levels of IL-6 and IL-8, which correlate with an enrichment in *Proteobacteria* and a decrease in the amount of certain butyrate-producing bacteria [51].

A recent paper reconstructs the longest human microbiota trajectory with age by phylogenetic GM analysis of a sizable number of Italian young, elderly and extremely long-lived subjects (centenarians and semi-supercentenarians, i.e., persons who reach the age of 105 years) [8]. According to the authors, a core GM comprised of dominant symbiotic bacterial taxa (*Ruminococcaceae*, *Lachnospiraceae*, *Bacteroidaceae*) loses diversity and relative abundance of its members with age, thus decreasing in size. In extreme longevity, this shrinkage is counterbalanced by an increase in longevity-adapted and possibly health-promoting subdominant species (e.g., *Akkermansia*, *Bifidobacterium*, *Christensenellaceae*) as well as in their co-occurrence network. In addition, the GM of semi-supercentenarians is invaded by micro-organisms typical of other niches, such as *Mogibacteriaceae* and *Synergistaceae*, known to be abundant in the periodontal environment. In extremely aged people, centenarians and semi-supercentenarians, an overall increase has been observed in the GM diversity. Thus, while extremely aged people lose some of the most important core components of the adult GM, they acquire in parallel a wealth of new microbial GM components, including potential pathobionts and allochthonous microorganisms. Along with extreme aging, it seems that the host tolerates the consolidation of new GM ecosystem balances in the gut, resembling a property typical of the ancestral human GM [54, 55]. In particular, to understand the GM-host’s co-evolutionary trajectory, several studies have been conducted comparing the GM ecosystem of small-scale rural societies and that found in a westernized lifestyle [54]. This comparison revealed specific GM adaptations to the respective subsistence strategies, including higher diversity and enrichment in microorganisms generally considered as pathobionts (e.g., *Prevotella*, *Treponema*, *Bacteroidetes* and *Clostridiales*) in the GM from ancestral populations [56, 57]. For instance, the GM of Hadza hunter–gatherers from Tanzania showed a unique enrichment in metabolic pathways that align with dietary and environmental factors peculiar to their foraging lifestyle, characterized by a broad-spectrum carbohydrate metabolism, reflecting the complex polysaccharides in their diet during the rainy season, though it is also equipped for the branched-chain amino acid degradation and aromatic amino acid biosynthesis typical of their diet during the dry season [55, 58]. Such research makes us appreciate the co-adaptive functional role of the GM in complementing human physiology.

Along with these studies, we can thus hypothesize that extremely long-lived people are able to rearrange the “mutualistic pact” with the GM, at least partly changing the microbial partners which support host health and physiology. It remains to be seen how these persons achieve this goal, and if and which environmental and/or genetic host factors are involved in this highly adaptive human process.

### Gut microbiota in centenarians from different continents: Italians versus Chinese and Japanese

In studying the age-related remodeling of the human GM, one of the most challenging aspects is to discriminate effects due to the aging process per se from those due to the modification of diet and lifestyle that aging entails [3]. In advanced age, tooth loss, chewing and swallowing problems, impaired sense of taste and smell and reduced physical activity strongly affect the quality of diet and lifestyle [59, 60] and these, in turn, are very well known short- and long-term determinants impacting on GM composition and functionality [3].

One effective strategy to disentangle these aspects is to compare elderly and long-lived people with different nutritional habits, lifestyles and cultures. Thus, comparison between GM of Italian centenarians/semi-supercentenarians and Chinese old people (including centenarians) led to identification of gut–microbial signatures during healthy aging [52]. The combination of the two datasets suggests significant differences in community membership and structures between the Italian and Chinese long-living groups that can be attributed to geographic, genetic and nutritional factors (Table 2). However, common features such as to discriminate long-lived from young people were identified in both groups [52]. Finally, the GM of the long-living groups in both the Italian and Chinese cohorts is also enriched in *Ruminococcaceae*, *Akkermansia* and *Christensenellaceae* which have been classified as potentially beneficial bacteria and linked to body mass index, immunomodulation and healthy homeostasis [52].

**Table 2:** Comparison of the changes to GM species composition between Italian, Chinese and Japanese centenarians according to different papers

				Change in Centenarians according to				
Phylum/class	Order/family	Genus (species)	Notes	Biagi et al. [51]	Biagi et al. [1]	Wang et al. [61]	Kong et al. [52]	Odamaki et al. [70]
*Actinobacteria/Actinobacteria*	*Bifidobacteriales/Bifidobacteriaceae*	*Bifidobacterium*		**↓**	↓ (100 +) ↑ (105 +)			
	*Micrococales/Microccocaeae*			**↑**				
*Actinobacteria/Coriobacteriia*	*Eggerthellales/Eggerthellaceae*	*Eggerthella (E. lenta)*		**↑**	↑ (105 +)			
*Bacteroidetes/Bacteroidia*	*Bacteroidales/Bacteroidaceae*				↓			
	*Bacteroidales/Odoribacteraceae*	*Odoribacter, Butyricimonas*			↑	**↓**		
	*Bacteroidales/Porphyromonadaceae*	*Parabacteroides*				**↓**		
*Firmicutes/Bacilli*	*Bacillales/Bacillaceae*	*Bacillus*		**↑**				
	*Bacillales/Staphylococcaceae*	*Staphylococcus*		**↑**				
	*Lactobacillales/Lactobacillaceae*	*Lactobacillus*				**↓**		
*Firmicutes/Clostridia*	*Clostridiales/Christensenellaceae*				↑ (105 +)		**↑**	
	*Clostridiales/Clostridiaceae*					**↑**		
	*Clostridiales/Clostridiales incertae sedis*	*Mogibacteriaceae*	Periodontal		↑ (105 +)			
	*Clostridiales/Eubacteriaceae*	*Eubacterium (E. limosum)*	*Clostridium* cluster XIVa	**↑**				
		*Eubacterium (E. hallii, E. ventriosum)*		**↓**				
	*Clostridiales/Lachnospiraceae*	*Blautia, Lachnobacterium (L. bovis, C. Sphenoides), Tyzzerella (C. Colinum)*		**↓**			↑ Lachnospiraceae (OTU173)	**↓**
		*Coprococcus*		**↓**	**↓**	**↓**		**↓**
		*Roseburia (R. intestinalis)*		**↓**	**↓**	**↑**		**↓**
		*unclassified Lachnospiraceae (E. rectale)*		**↓**				
	*Clostridiales/Ruminococcaceae*	*Anaerotruncus (A. colihominis)*	*Clostridium* cluster IV	**↑**	↑ (105 +)		↑ Ruminococcaceae (OTU018)	
		*Faecalibacterium (F. prausnitzii)*		**↓**	↓	↓		↓
		*Oscillospira*			↑	↑ OTU411, ↓ OTU 163		
		*Papillibacter (P. cinnamovorans)*		**↓**		↑ OTU316, OTU321, OTU69, OTU201, OTU598, OTU388; ↓ OTU732, OTU347, OTU616, OTU427		
		*Ruminiclostridium (C. Leptum)*		**↑**				
		*Sporobacter (S. termiditis)*		**↑**				
*Firmicutes/Negativicutes*	*Selenomonadales/Selenomonadaceae*	*Megamonas, Mitsuokella*				**↓**		
*Fusobacteria/Fusobacteriia*	*Fusobacteriales/Fusobacteriaceae*	*Fusobacterium*		**↑**				
*Proteobacteria/Betaproteobacteria*	*Burkholderiales/Sutterellaceae*	*Sutterella*				**↓**		
*Proteobacteria/Deltaproteobacteria*	*Desulfovibrionales/Desulfovibrionaceae*	*Bilophila*			↑ (105 +)			
*Proteobacteria/Gammaproteobacteria*	*Enterobacteriales/Budviciaceae*	*Leminorella*		**↑**				
	*Enterobacteriales/Enterobacteriaceae*	*Escherichia (E. coli), Klebsiella (E. aerogens, K. Pneumoniae)*		**↑**		**↑**		**↑**
	*Enterobacteriales/Morganellaceae*	*Proteus*		**↑**				
	*Enterobacteriales/Yersiniaceae*	*Serratia, Yersinia*		**↑**				
	*Pasteurellales/Pasteurellaceae*	*Haemophilus*		**↑**				
	*Pseudomonadales/Pseudomonadaceae*	*Pseudomonas*		**↑**				
	*Vibrionales/Vibrionaceae*	*Vibrio*		**↑**				
*Synergistetes/Synergistia*	*Synergistales/Synergistaceae*		Periodontal		↑ (105 +)			
*Verrucomicrobia/Verrucomicrobiae*	*Verrucomicrobiales/Verrucomicrobiaceae*	*Akkermansia*		**=**	↑ (105 +)	**↓**		

Another paper, presenting the Illumina sequencing of 16S rRNA gene amplicons performed on the GM of centenarians living in one of the most long-lived villages in the world (Bama County, China), confirmed that the GM of centenarians was more diverse (count of the unique OTU numbers, Chao 1 index) than that of the younger elderly [61] (Table 1). The diversity of the GM community is considered as a key health indicator since it markedly affects the health status of the hosts, while a reduced GM diversity has been associated with several pathological conditions, including autoimmunity (inflammatory bowel disease and psoriatic arthritis), antibiotic treatment, *Clostridium difficile* infections, obesity and other metabolic alterations [62–69]. These results contrast with previous studies suggesting that the microbial diversity of GM was significantly reduced in centenarians [51]. However, in Biagi et al. [51] the GM was characterized by a microarray-based approach, making it impossible to fully characterize any unexpected diversity of the human GM ecosystem. Among the distinctive features of the fecal microbial communities of Bama County centenarians, the authors showed certain similarities (abundance of *Escherichia*, reduction in *Bacteroidetes*, structural change in butyrate-producing bacteria in the *Clostridium* cluster IV and *Clostridium* cluster XIVa) and some differences (low level of *Akkermansia*) with Italian centenarians (Table 2).

The paper by Odamaki et al. [70] provides a picture of the changes in the GM composition throughout human life, from birth to extreme aging in a large cohort of Japanese individuals [70]. However, even though children, adults and the elderly were abundantly represented, this analysis was not centered on longevity, including only six centenarians (100–104 years old) and seven over-95 year-olds. Importantly, a decrease in *Faecalibacterium*, *Roseburia*, *Coprococcus*, *Blautia* and an increase in *Enterobacteriaceae* were shown in 90- and 100-year-old subjects, resembling the age-related microbiota features found in Italian centenarians but with some differences from Chinese centenarians (Table 2). Regarding the microbiota diversity, in the Japanese cohort the alpha diversity score and the Shannon index remained stable during adulthood and then increased in the elderly and centenarians, the later data confirming previous observations (Table 1) [70].

GM remodeling with age matches metabolome variations. Thus, centenarians showed a distinct metabolic pattern. A unique alteration of specific glycerophospholipids and sphingolipids [71] and decreased circulating levels of 9-hydroxy-octadecadienoic acid (9-HODE) and 9-oxo-octadecadienoic acid (9-oxoODE), markers of lipid peroxidation [7], are seen in the longevity phenotype in Italy. It has also been revealed that the longevity process deeply affects the structure and composition of the human GM-derived metabolome, as shown by the increased excretion of phenylacetylglutamine (PAG) and p-cresol sulfate (PCS) in Italian centenarians’ urine [7]. In 647 individuals from the US, followed up for as much as 20 years, higher concentrations of the citric acid cycle intermediate, isocitrate, and the bile acid, taurocholate, were associated with lower odds of longevity, defined as attaining 80 years of age. In a larger cohort of 2327 individuals with metabolite data available, higher concentrations of isocitrate but not taurocholate were also associated with worse health conditions [72]. On the other hand, centenarians from the Bama County in China showed decreased levels of PCS but increased levels of fecal short-chain fatty acids (SCFAs) and total bile acids [73]. Intestinal commensal bacteria metabolize host-derived bile salts [74]. Bile acids are hormones that regulate their own synthesis, transport, glucose and lipid homeostasis and energy balance via activation of specific nuclear receptors and G protein-coupled receptors. The circulating bile acid pool composition consists of primary bile acids produced from cholesterol in the liver, and secondary bile acids formed by specific gut bacteria. The gut microbial community, through its capacity to produce bile acid metabolites distinct from the liver (i.e., secondary bile acids), can be thought of as an “endocrine organ” with the potential to alter host physiology, perhaps in their own favor. The term “sterolbiome” [74] describes the genetic potential of the GM to produce endocrine molecules from endogenous and exogenous steroids in the mammalian gut. Thus, changes in age-associated microbiome composition could impact on bacterial metabolism of steroid compounds and ultimately steroid hormones in peripheral tissues. Chinese centenarians have high levels of bile acids [73], suggesting a pro-longevity role. However, studies on different populations reported that increased levels of secondary bile acids are associated with an increased risk of age-associated diseases [72] and specific diseases of the gastrointestinal tract system [75].

On the whole, these data indicate that human GM alterations during aging are not univocal (Table 2) but follow different trajectories depending on lifestyle, nutrition, geographic/population/social factors as well as host genetics. In extremely long-lived people the composition, functionality and diversity of this complex and dynamic microbial community seem to achieve a peculiar balance resulting from a continuous 100-year remodeling process. Thus, it still remains to be determined how and if this (optimally?) adapted GM contributes to the homeostasis of the aged host, enabling him/her to reach the extreme limits of human life.

## Gut microbiota age-related changes, brain functions and neurodegenerative diseases

**Fig. 1:**
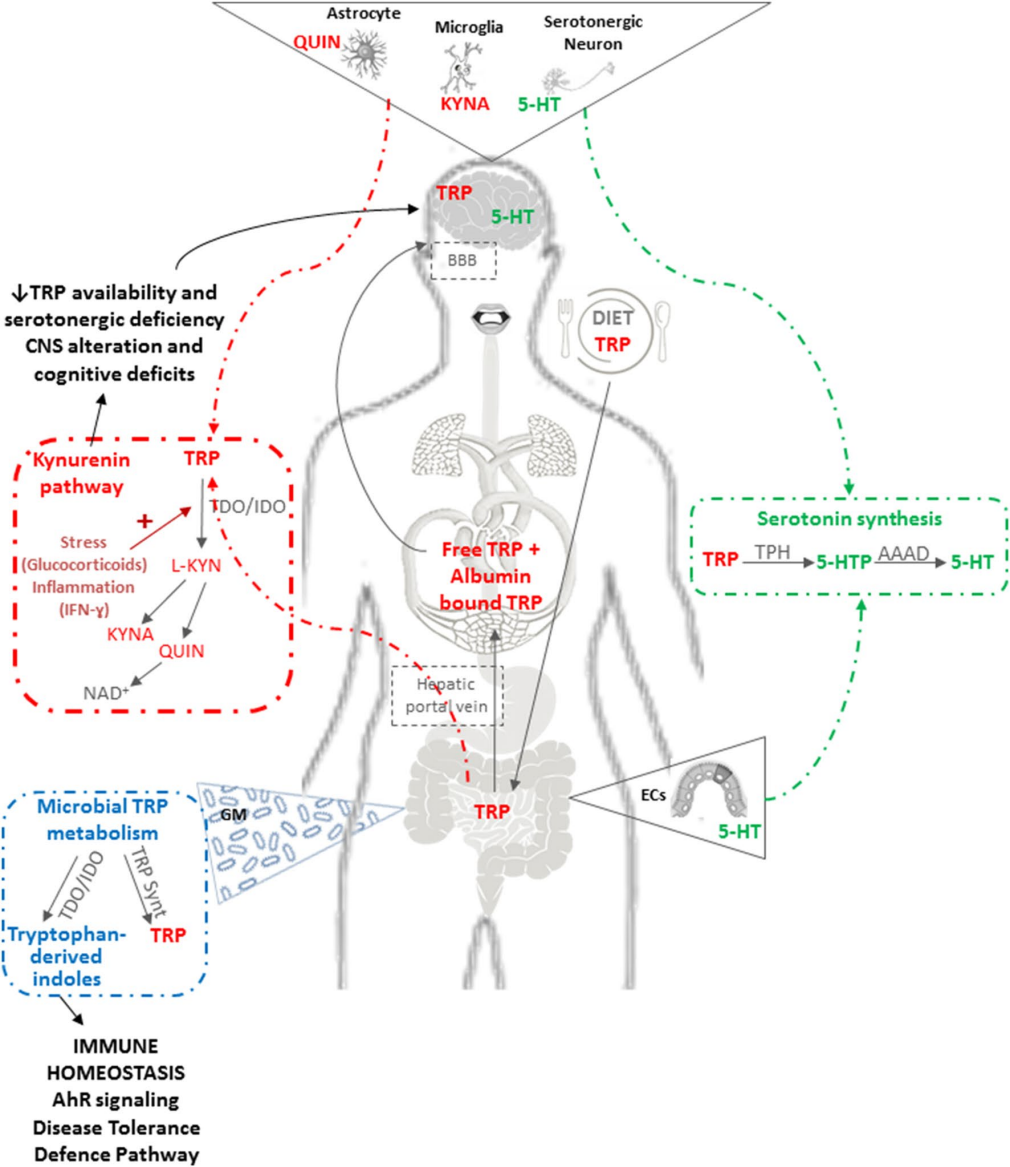
Tryptophan metabolism through the serotonin and kynurenine pathway. Tryptophan (TRP) is an essential amino acid which must be supplied with the diet. Once absorbed from the gut, TRP is made available in circulation as free TRP and albumin-bound TRP fraction and/or is metabolized along the serotonin or the kynurenine pathway. TRP in circulation can cross the blood–brain barrier (BBB) to participate in serotonin (5-HT) synthesis in the CNS. TRP in the gut is metabolized to 5-HT in the enterochromaffin cells (ECs): TRP is first converted to 5-hydroxytryptophan (5-HTP) by the rate-limiting enzyme tryptophan hydroxylase (TPH), then the short-lived 5-HTP intermediate product is decarboxylated to 5-HT by aromatic amino acid decarboxylase (AAAD). However, the vast majority of available TRP is metabolized along the kynurenine pathway. Kynurenine (L-KYN) is produced from TRP by the action of the hepatic enzyme, tryptophan-2,3-dioxygenase (TDO) or the ubiquitous indoleamine-2,3-dioxygenase (IDO). TDO can be induced by glucocorticoids or by TRP itself, whereas IDO is stimulated by inflammation with IFN-ɣ as the most potent inducer. Once L-KYN is produced, it is further metabolized along one of two distinct arms of the pathway with the production of neuroprotective kynurenic acid (KYNA) or neurotoxic quinolinic acid (QUIN). KYNA can be neuroprotective against QUIN-induced excitotoxicity but it can also induce cognitive impairment when abnormally elevated. Activation of the kynurenin pathway has a dual impact by limiting the availability of TRP for 5-HT synthesis and increasing the downstream production of neurotoxic/neuroprotective metabolites. TRP, via the kynurenine pathway, is involved in the biosynthesis of nicotinamide adenine dinucleotide (NAD^+^) which is an essential coenzyme for cellular processes of energy metabolism, cell protection and biosynthesis. The GM can also directly utilize TRP, limiting its availability to the host. Certain bacterial strains may produce a tryptophanase enzyme that synthetizes indoles from TRP. These microbial metabolites have recently been identified as human aryl hydrocarbon receptor (AhR)-selective agonists. AhR signaling has a role in chemical/microbial defense and tissue development, while, recently, IDO–AhR axis has been recognized as a fundamental player in controlling the “Disease Tolerance Defense Pathway”. Bacteria can also synthesize tryptophan via enzymes such as TRP synthase (TRP synt) and specific bacterial strains can also produce serotonin from TRP in vitro. The balance between bacterial TRP utilization and metabolism, TRP synthesis and 5-HT production plays an important role in regulating gastrointestinal and circulating TRP availability for the host in addition to its dietary intake. Moreover, accumulating evidence supports the role of the GM in regulating TRP availability and 5-HT synthesis via modulation of the enzymes responsible for TRP degradation along the kynurenine pathway

To this already complex scenario, it should be added that the gastrointestinal tract establishes a strong bidirectional connection with the Central Nervous System (CNS) named the “gut–brain axis”, along which the GM plays a crucial role. A number of experimental observations have shown that even mild alterations in GM composition are able to cause modification of cerebral functions, while conversely the brain can deeply affect intestinal functions via the secretion of hormones, neuropeptides and neurotransmitters such as substance P, neurotensin, corticotropin releasing hormone, 5-hydroxytryptamine, and acetylcholine. The literature on this hot topic is extensive and more details can be found in recent reviews [76, 77]. Here, specific topics relating to the impact of GM age-related changes on brain physio-pathology, with particular attention to the role of tryptophan, will be briefly addressed. Gut and the GM affect brain and upper cognitive functions by two distinct pathways: (1) a direct one via retrograde stimulation of the Vagus nerve and the production of hormones and cytokines such as IL-6, TNF-α and VIP; (2) an indirect one, via the production of bacterial components and metabolites. The main microbial bioactive molecules are: proteins that may cross-react with human antigens and stimulate abnormal responses by the immune system [78, 79]; neurotoxic metabolites such as d-lactic acid and ammonia which are able to cross the blood–brain barrier and cause neurotoxicity or neuroinflammation [80–82]; hormones and neurotransmitters interfering with those of human origin (e.g., *Lactobacillus* and *Bifidobacterium* species are GABA neurotransmitter producers, *Escherichia*, *Streptococcus* and *Enterococcus* are serotonin synthesizers) [83–85]. Hence, instead of a “gut–brain axis”, it would be more correct to refer to the “GM–gut–brain axis” integrating the GM with neuro-humoral signals from/to the CNS, neuroendocrine and immune systems, the autonomic nervous system, and the enteric nervous system (ENS). A growing amount of evidence has pinpointed the availability and metabolism of the essential amino acid tryptophan as a key regulator of this axis. Tryptophan is metabolized along the serotonin or the kynurenine pathway [86] with many implications for ENS and CNS functioning (Fig. 1). Serotonin is mainly (95%) located within the GI tract and in a small proportion (5%) in the CNS. In the gastrointestinal tract serotonin is responsible for motility, secretion and absorption as well as intestinal transit, while it can also modulate food intake by stimulating vagal afferent pathways involved in the reduction of obesity and metabolic dysfunction [87]. By contrast, most available tryptophan is transformed into quinolic and kynurenic acid, which are of particular interest for neurogastroenterology as they are neuroactive metabolites that act on *N*-methyl-d-aspartate (NMDA) and alpha 7 nicotinic acetylcholine receptors in the CNS and ENS. In the CNS, kynurenic acid has long been viewed as neuroprotective, whilst quinolinic acid is primarily considered an excitotoxic NMDA receptor agonist [88]. Within the gastrointestinal tract, both molecules appear to be involved in immunoregulation [89] and in particular kynurenic acid may have anti-inflammatory properties [90]. Due to its specific role on tryptophan metabolism and serotonergic system, there is some evidence that the GM is a pivotal player in the regulation of different behavioral domains such as pain, depression, anxiety and cognition [86]. Major studies about this relationship have been performed using germ-free animals (free of all microorganisms, including those normally symbiotic in the gut) characterized by increased plasma tryptophan concentrations that can be normalized by colonizing the mice immediately post-weaning. These animals exhibited increased hippocampal 5-hydroxytryptophan (a serotonin precursor) concentration and significant CNS alterations, demonstrating that the GM is essential for normal brain development [91, 92]. In fact, the GM can directly utilize tryptophan, limiting its availability to the host because bacteria require tryptophan for their normal growth and some strains such as *Bacteroides fragilis* may produce a tryptophanase, an enzyme that has recently been associated with autism spectrum disorders [93] (Fig. 1). Moreover, some bacterial strains are able to synthesize tryptophan and produce serotonin from tryptophan in vitro. Tryptophan, through the kynurenine pathway, is involved in the biosynthesis of nicotinamide adenine dinucleotide (NAD^+^) [94], which has a key role in human health as it is an essential coenzyme for the cellular processes of energy metabolism, cell protection and biosynthesis. Moreover, decreased cellular NAD^+^ concentrations occur during aging and supplementation with NAD^+^ precursors can prolong both life span and health span [95, 96]. NAD^+^ is indeed an important co-substrate of sirtuins. Several papers have shown that in old animals, when the levels of NAD^+^ are restored, there is an increase in sirtuin1 and a reduction in mitochondrial stress, DNA damage and inflammation [95].

Tryptophan-derived indoles are involved in the host–microbiome interaction in the intestine [97]. Indoleamine-2,3-di-oxygenase (IDO) is an interferon-γ-induced enzyme involved in catabolizing tryptophan to kynurenine, which has been shown to be higher in nonagenarians than in young people [98]. Hence, inflammaging might induce IDO, leading to tryptophan degradation to kynurenine. Microbial tryptophan metabolites generated by induction of IDO have recently been identified as human aryl hydrocarbon receptor (AhR)-selective agonists [99]. AhR signaling has a role in various physiological processes including chemical/microbial defence and tissue development, while, recently, the IDO–AhR axis has been recognized as a fundamental player in the control of the “Disease Tolerance Defence Pathway”, i.e., the ability of the host to reduce the effect of infection on host fitness [100] (Fig. 1). Data obtained on murine models have shown that tryptophan catabolism by IDO assumes an immunoregulatory role acting via AhR ligands, boosting regulatory T cells and protecting mice from chronic hyperinflammatory responses [101].

The balance between bacterial tryptophan utilization, metabolism and synthesis and serotonin/kynurenine production has a fundamental function in determining local gastrointestinal and circulating tryptophan availability for the host with implications for both ENS and CNS serotonergic neurotransmission [86].

**Fig. 2:**
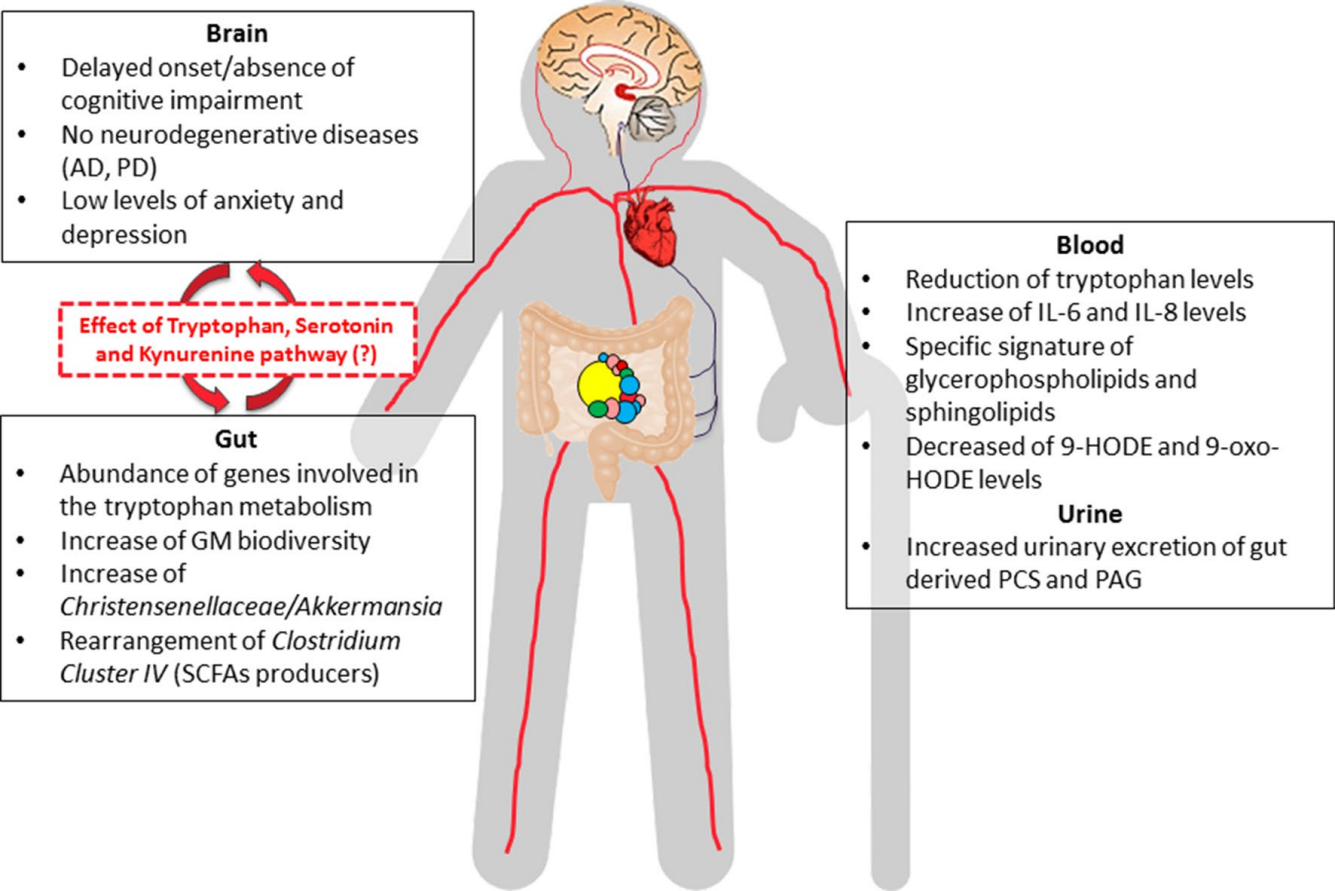
Gut microbiota and brain function in Italian centenarians. This figure summarizes our studies on the phenotypic characteristics of Italian centenarians. In extreme longevity complex remodeling of the GM is reflected at a systemic level by specific signatures of blood and urine markers (inflammatory, lipidic and metabolic). The strong two-way connection between GM and brain is likely to positively affect the well-preserved cognitive function of centenarians until a very advanced age. The fundamental role in the effect on the brain by bacterial tryptophan metabolism via the serotonin and/or kynurenine pathways deserves to be further investigated. *AD* Alzheimer’s disease, *PD* Parkinson’s disease, *SCFAs* short-chain fatty acids, *IL-6* interleukin-6, *IL-8* interleukin-8, *9-HODE* 9-hydroxy-octadecadienoic acid, *9-oxo-HODE* 9-oxo-octadecadienoic acid, *PCS* p-cresol sulfate, *PAG* phenylacetylglutamine

Modifications to the composition of GM across the lifespan may deeply affect the availability of tryptophan and serotonergic signaling during aging. Shotgun analysis on the bacterial metagenome of young, old and centenarian subjects showed an age-related amplified abundance of genes involved in the tryptophan metabolism pathway [102] and this finding is in agreement with the reduction of tryptophan due to its altered bio-availability found in the serum of centenarians [7, 71]. Little is known about plasma tryptophan disposition in aged experimental animals, while in humans the plasma concentration of tryptophan is moderately lower in the elderly [103]. It is interesting to note that in rodents, limiting the dietary intake of tryptophan and methionine may have a beneficial effect on health- and life span [104], while excess of tryptophan can be toxic and carcinogenic [105]. In addition, alteration of the kynurenine metabolites may contribute to neurotoxicity [106] and has been associated with Huntington disease [107], HIV dementia [108] and Parkinson’s disease (PD) [109]. Surprisingly, in a mouse model of Alzheimer’s disease (AD), a diet rich in tryptophan seems able to reduce the amyloid plaque content [110]. It is thus tempting to speculate that the GM of centenarians adjusted the tryptophan metabolism to support healthy aging (Fig. 2). These findings assume a particular importance in view of the fact that centenarians are remarkably free from neurodegenerative pathologies such as PD and AD. Indeed, although the prevalence of cognitive impairment in centenarian studies varies widely [111], some of them (15–20%) preserve cognitive function and, even among those who show cognitive impairment at 100 years, approximately 90% delay the onset of clinically evident dementia until the advanced average age of 92 years [112]. In addition, most centenarians have low levels of anxiety and depression [111], suggesting that such people should be chosen as “super-controls” in studies designed to evaluate the contribution of GM dysbiosis to cerebral degenerative diseases.

AD is one of the commonest neurodegenerative disorders and associates with cerebral accumulation of amyloid-beta fibrils driving neuroinflammation and neurodegeneration. The bacterial species residing in the intestine have been shown to release substantial amounts of amyloids and lipopolysaccharides, thereby promoting the production of pro-inflammatory cytokines and modulating the signaling pathways involved in the pathogenesis of AD [113, 114]. Numerous research findings have shown that AD may start in the gut, and hence is closely associated with GM imbalance. There is increasing evidence to suggest a link between GM and PD. Recent studies showed that PD is associated with gut dysbiosis [115]; the fecal concentration of SCFAs is significantly reduced in PD patients compared to controls and this reduction could impact on CNS alterations and contribute to gastrointestinal dysmobility in PD [116]. In a mouse model of PD, it has been demonstrated that GM is a key player in motor deficits and microglia activation [117]. Interestingly, alpha-synuclein aggregates, a pivotal marker of PD, are present in both the submucosal and myenteric plexuses of the ENS, prior to their appearance in the brain, indicating a possible gut to brain route of “prion-like” spread [118].

The GM role has also been investigated regarding regulation of hypothalamic–pituitary–adrenal (HPA) axis development [119, 120]. In germ-free mice, exposure to a restraint stress triggers an exaggerated HPA axis response, as compared to specific pathogen-free control mice. Such an aberrant response is normalized through intestinal colonization by *Bifidobacterium longum* subsp*. infantis*, and fecal matter from specific pathogen-free mice. Importantly, fecal microbiota transplantation (FMT) proved efficient only in animals’ early life [121]. These experiments demonstrated the crucial function of the GM in the development of an appropriate physiological endocrine response versus stress in the postnatal stage of the animal model. During life, chronic HPA axis hyperactivation by stress exposure damages the gut barrier integrity, causing intestinal dysbiosis, behavioral changes and stress-related symptoms, including mood disorders, anxiety and cognitive defects [122]. Patients suffering from hepatic encephalopathy are characterized by alterations of GM composition and endotoxemia. In particular, high levels of inflammatory cytokines were found in cirrhotic patients with cognitive decline, as compared to those with normal cognitive function, and the bacterial families *Alcaligeneceae* and *Porphyromonadaceae* proved positively correlated with cognitive impairment [123, 124]. Other works have focused on the impact of the GM on depression or anxiety, showing that pathogen-free mice exhibit reduced anxiety and increased motor activity [91, 125]. Tillisch et al. [126] demonstrated that brain activity and connectivity in healthy women following an emotive task could be attenuated by administering a 4-week course of a fermented milk beverage containing several probiotic bacterial strains [126]. Thus, the GM seems to modulate multiple effects, overcoming even the adaptive immunity functions besides revealing neurological/psychological potential. The two-way interaction between GM and the brain can be modulated by diet and/or probiotic/prebiotic/symbiotic supplementation designed to positively impact on brain activity and behavior [127]. For these reasons, probiotics with psychotropic functions in humans, such as *Lactobacillus helveticus* and *Bifidobacterium longum*, have recently been termed “psychobiotics” given their ability to reverse anxiety or depression-like behavior [128].

### Gut microbiota-targeted diets and interventions improving cognition and health

The marked potential effect of the GM on neurological and psychological pathways suggested the hypothesis that intestinal bacteria may be a bridge in the emerging relation between diet and the cognitive system [123]. For example, pronounced consumption of fruit, vegetables and pulses typical of the Mediterranean Diet (MedDiet) has been associated with increasing fecal SCFA levels. SCFAs (acetate, propionate and butyrate), produced by GM (*Firmicutes* and *Bacteroidetes* strains) during fermentation of undigested polysaccharides, has a well-documented protective role on various inflammatory as well as behavioral disorders [129, 130].

It has recently been shown that the GM rapidly responds to altered diet in a diet-specific manner. It seems possible to modulate GM composition and activity within a single day, switching from herbivorous to carnivorous diet and as a consequence modulating GM metabolic pathways [131]. Thus, the dietary lifestyle represents a long-life stimulus for the GM, which responds by modifying its structure and functionality in the short term with multiple effects on the organism.

Recently, it has been postulated that the MedDiet exerts its health effects through hormetic mechanisms [132]. A lifelong exposure to the specific components of the MedDiet may, therefore, very likely counteract the effects of inflammatory stimuli, including those that may come from the GM metabolism, by acting as hormetins [132]. Epidemiologic evidence also suggests that coffee drinkers have a lower risk of PD [133]. It has been proposed that this protective effect impacts on the composition of the GM, counteracting the development of intestinal inflammation which is associated with less misfolding of the protein alpha-synuclein in the enteric nerves. This would reduce the risk of PD development, minimizing propagation of the alpha-synuclein aggregates to the CNS [118].

In animal models, interventions aimed at reducing calorie intake have been shown to be accompanied by structural modulation of the GM [134]. For instance, a life-long low-fat diet significantly altered the overall structure of the GM in C57BL/6J mice. Calorie restriction was shown to enrich phylotypes positively correlating with longevity, such as the genus *Lactobacillus*, and to reduce phylotypes negatively associated with lifespan [135]. Since nutrient metabolism is highly dependent on the composition of the GM and vice versa [136], it can be assumed that certain anti-aging interventions may cause specific variations to gut microbial communities causing chronic calorie restriction conditions and thus promoting both the health span and the life span. Several documented clinical trials have investigated the effect of prebiotics and probiotics, particularly those containing *Bifidobacterium* and *Lactobacillus*, as a microbiota-targeted intervention to improve health status in elderly populations [137–140]. Most of the benefits are mediated by the activation of anti-inflammatory pathways in the residents’ microorganisms. Probiotic supplementation may also improve metabolic and cardiovascular health status [141] and promote longevity by stimulating the innate immune response [142, 143], improving resistance to oxidative stress [144], decreasing lipofuscin accumulation [145] and modulating serotonin signaling [146]. There is also evidence that probiotic treatment can promote longevity in mice, possibly through suppression of chronic low-grade inflammatory processes in the colon [147]. Importantly, several findings suggest that direct modulation of the GM may not only be applied in treating particular age-related disorders, but can also be a promising therapeutic option to combat the aging process per se. For example, in a murine model, oral administration of purified exopolysaccharide fractions from *Bifidobacterium animalis* RH that were isolated from the fecal samples of centenarians residing in Bama longevity villages (Guangxi, China) resulted in significantly increased activity by superoxide dismutase, catalase and total antioxidant capability in serum, as well as reduced levels of lipofuscin accumulation in the mouse brain [148].

Another approach to restoring the intestinal ecosystem is FMT, also called bacteriotherapy, a transfer of liquid filtrate feces from a healthy donor into the recipients’ gastrointestinal tract to treat a particular disease or condition [149]. Initially, bacteriotherapy was developed as an effective method of treating *Clostridium difficile* infection, which is a major cause of healthcare-associated diarrhea through perturbation of the normal GM [150]. More recently, its potential effectiveness and safety has been hypothesized in the prevention and treatment of non-gastrointestinal pathologic conditions, including those commonly associated with aging, e.g., atherosclerosis, metabolic syndrome, type 2 diabetes and neurodegenerative diseases [151, 152]. In a preliminary study of the effectiveness of FMT in humans, transferring GM from lean donors to persons with metabolic syndrome [153] beneficially affected the GM composition in recipients by increasing amounts of butyrate-producing bacteria along with improved insulin sensitivity 6 weeks after the FMT procedure [154]. Improvements in symptoms of PD in patients receiving FMT were described in one case report [155], while no studies have been reported for AD so far.

In this scenario the knowledge emerging from GM studies in centenarians may soon be exploited for therapeutic purposes. For example, transplantation of centenarians’ GM into germ-free animal models will allow us to identify the bacteria or bacterium combination that could be protective against neurodegenerative diseases.

## Gut microbiota and host genetics: an intimate evolutionary-shaped relationship

During the last few years, an impressive amount of literature has been published on the different strategies to modify and improve the GM diversity structure with a view to promoting human health. Similarly, many pathologies ranging from obesity and inflammatory diseases to behavioral and physiological abnormalities with neurodevelopmental disorders have been associated with different types of bacterial species and their products [77], as described in the previous sections.

On the other hand, recent data have suggested a new and intriguing possibility that the host genome interacts and shapes its own GM. In this connection host genetics have been shown to influence the composition of the GM in twin studies [156, 157], while more recently in a wider population study, *Christensenellaceae* have been reported as the chief bacterium family associated with genetics [158]. The abundance of *Christensenellaceae* was also associated with lower body mass index (BMI) in twins, and when introduced into a mouse model it led to reduced weight gain in treated mice compared with controls [158], suggesting that the microbiome can be an important mediator between host genetics and phenotype. Intriguingly, these bacteria were found to characterize the GM in extreme longevity [8], thus reinforcing the idea of a close association with the genetic background and suggesting a possible link to the inheritable component of human longevity. Nuclear, also mitochondrial, DNA plays a major role in the aging process so the complex interaction between these two host genetics [159] should be taken into account if we are to properly address the GM remodeling occurring during the human life span.

The intimate symbiotic relationship between host genetics and the GM is very ancient since vertebrates coevolved along with their gut bacteria. Multiple lineages of the predominant bacterial taxa such as *Bacteroidaceae* and *Bifidobacteriaceae* in the gut arose via co-speciation within hominids over the past 15 million years [160]. The divergence times also indicate that nuclear, mitochondrial, and gut bacterial genomes diversified in concert during hominid evolution [160]. Interestingly, it seems that gut microbiomes have recorded the information of major dietary shifts that occurred during the evolution of mammals, allowing us to predict ancient diets from the reconstruction of ancient microbiomes [161].

Recently, genome-wide association screening for host genetic associations with GM composition identified 42 loci (mainly related to innate immunity) associated with GM variation and function in humans [162]. Another study identified significant associations between gut microbial characteristics and the VDR gene (encoding vitamin D receptor), in addition to a large number of other host genetic factors, and eventually quantified the total contribution of host genetic loci to diversity as 10.43% [163]. The non-genetic factors such as age, sex, BMI, smoking status and dietary patterns explain 8.87% of the observed variations in the GM [163]. Even though the effect of individual genes is small and comparable with the cumulative effect of key non-genetic covariates, the underlying biology of these studies provides a critical framework for future assessments of host–microbe interactions in humans with an adequate statistical power and sample size. Associations with gut microbial community composition at the VDR locus provide a link with secondary bile acids, which serve as ligands for VDR. Results from gene set enrichment analysis and the observation that the bile acid profile in serum associates with variation in the gut microbiome [163] further support this finding. A detailed description of the effect of host genetics on GM composition lies outside the scope of this review. Kurilshikov and colleagues recently published a comprehensive summary of the state of the art on host genetic determinants of GM with details as to techniques and populations analyzed, to which readers are referred [164].

A recent bioinformatics analysis predicts that long non-coding RNAs expressed in the intestinal epithelial cell in murine models constitute molecular signatures reflecting the different types of microbiome [165]. In this direction, very recent data highlight the role of the host genome in shaping the GM, even if in terms of microRNAs (miRs). MiRs produced by gut epithelial cells enter bacterial membrane, modifying bacterial gene expression in in vitro models [166]. In a mouse model (DICER deficiency), a severe dysbiosis develops when miR maturation is deficient. These important findings not only outline the tight coevolution and inter-organismal crosstalk leading to various profound cellular and metabolic changes, but also lay the foundations for new miR-based therapies to counteract gut-related diseases.

Many variables may be responsible for GM remodeling associated with human longevity. Among these, the genetic makeup of extreme longevity [159, 167], and the epigenetic changes associated with aging could have a deep impact together with nutrition and lifestyle habits. These lifelong interactions by variables are expected to have significant outputs in the production of specific blood/urine biomarkers or longevity-associated metabotypes. This is the case with centenarians. As reported above, Italian centenarians show increased excretion of bacterial products such as PAG and PCS in urine [7], specific blood lipid profiles and changes in amino acid levels [7, 71] (Fig. 2). By contrast, centenarians from the Bama County in China showed decreased levels of PCS and increased levels of fecal SCFAs and total bile acids [73]. All these findings support the hypothesis of a complex remodeling of the lipid and amino acid metabolism correlated with GM changes [7], as a result of lifelong adaptation and coevolution processes that could also be ethnic specific. Of note, it still remains to be clarified what role gender plays in GM modification studies on long-lived subjects, since female centenarians outnumber males. A much deeper knowledge of the relationship between host genetics and the GM emerged from a recent paper, which used shotgun analysis on 250 adult twins from the UK [168]. These data showed that GM composition and functions are inheritable and that twin pairs share microbial SNPs. Interestingly, this similarity is lost after decades of living apart [168], emphasizing the impact of household and geographic region on the GM.

### Lifelong interaction among sex, sex hormones and gut microbiota

Several studies have shown that sex hormones also play a role in the host–microbiota interaction. Indeed the term “microgenderome” defines the potential mediating and modulatory role of sex hormones on GM function and composition with implications for autoimmune and neuroimmune conditions [169]. Sexual dimorphism is common in autoimmune diseases. Using the non-obese diabetic mouse model of Type 1 Diabetes, Markle et al. showed that the gut commensal microbial community strongly conditions the pronounced sex bias in Type 1 Diabetes risk by controlling serum testosterone and metabolic phenotypes [170]. Their results revealed evidence of sex-specific microbial communities and sex-specific responses to the same microbial communities. The same group also found that the recipients’ GM was stably altered in a sex-specific way, since male-typical changes in the GM of female recipients were evident for several months. Unexpectedly, these experimental GM manipulations strongly protected the female mice from diabetes. The mechanism behind this protection critically depended on the impact of the GM on host metabolism and sex hormone signaling pathways [171]. A number of different taxa have been found between male and female mice, while the sex differences in GM composition depend in part on genetic background [172]. Using gonadectomized and hormone-treated mice clearly revealed hormonal effects on the GM composition [172]. In humans, sex-specific interactions between *Firmicutes* and neurological, immune and mood symptoms of myalgic encephalomyelitis/chronic fatigue syndrome have been reported [173], but we are just beginning to appreciate the links between human microbiome composition and hormonal phenotypes. Twin studies have revealed that the once similar microbial composition of opposite-sex twins becomes distinctly different after puberty when compared to that of same-sex twins which remains compositionally similar [57]. These data suggest that age-specific interactions of the host with specific microbes may exert beneficial and/or detrimental influences on the biology of the host, including either protection from or susceptibility to autoimmune disease. Furthermore, microbiota transfer studies in humans, mice, and rats reveal a high degree of host specificity on the part of the GM. Bacterial gene expression modulation by the host may partly explain the failure of FMT in certain specific cases, such as those related to *Clostridium difficile* infection treatment [174] and eventually impact on GM remodeling with age [8]. Efficient colonization and associated effects also seem to be most successful in young animals, most likely because their microbiota is not yet stabilized [169].

Dietary effects on the composition and diversity of GM depend in part on sex-specific interactions [172, 175]. An interesting work showed that GM composition depends on interactions between host diet and sex within populations of wild and laboratory fish, laboratory mice and humans. The inter-individual diet variation correlates with individual differences in the GM and these diet–microbiota associations are sex dependent. In mice, experimental diet manipulations confirmed that diet affects the GM differently in males versus females. Thus, the prevalence of the individual genotype interacting with the environment (e.g., sex by diet) implies that therapies to treat dysbiosis might have sex-specific effects [176].

## Conclusions

Overall, the data available on lifelong changes in the GM are still too few for us to draw any definitive conclusions as to the basic question of how much can be set down to variables such as population, diet, genetics and gender, and how much to the aging process per se. In particular, the GM changes occurring in the last two or three decades of life (in nonagenarians, centenarians, semi-supercentenarians and supercentenarians, i.e., persons who reach the age of 110 years) have been insufficiently investigated, especially regarding the possible contribution of GM to health and longevity or to cognitive decline and neurodegeneration. Longitudinal studies envisaging metagenomics sequencing and in-depth phylogenetic analysis as well as an extensive phenotypic characterization using up-to-date omics (metabolomics, transcriptomics and meta-transcriptomics, to mention a few) are urgently needed. The results of this comprehensive approach are likely to offer more satisfactory answers to the questions addressed in this paper.
